# The emergency (crisis) e-learning as a challenge for teachers in Poland

**DOI:** 10.1007/s10639-021-10539-7

**Published:** 2021-04-17

**Authors:** Łukasz Tomczyk, Christopher Walker

**Affiliations:** 1 Pedagogical University of Cracow, Ingardena 4 street, 30-060 Kraków, Poland; 2 International House Bielsko-Biała, Bielsko-Biała, Poland

**Keywords:** E-learning, E-learning crisis, Teachers, Poland, Challenges, Problems, Digital competence, COVID, Pandemic

## Abstract

The article was written as a consequence of the COVID-19 pandemic in Poland, which had an impact not only on public health, but also on the functioning of the educational sector. The text is an attempt to summarize the challenges of crisis e-learning from the perspective of the challenges faced by teachers in Poland in the period of March-December 2020. The article reveals a number of new phenomena not present in the literature in the context of e-learning implemented in an intuitive, non-linear way, without methodological support, and thus referred to as crisis e-learning. The aim of the research was to explore the characteristics of crisis e-learning in Poland from the perspective of teachers' experiences. Due to epidemiological limitations, the research area was narrowed down to cyberspace. This text presents the results of research relating to statements made by teachers posting in the largest Polish discussion group on education. The group currently consists of over four thousand people. The study uses an analysis of several thousand posts and then identifies and categorizes statements related to crisis e-learning along with a phenomenological interpretation. The analyses made it possible to identify seven categories of challenges attributed to crisis e-learning, such as: technical problems, use of non-standard solutions, the search for solutions to increase the effectiveness of e-learning, the transfer of proven applications and programmes, problems with students, problems with parents, and the modernisation of workstations. The data presented show teacher micro-worlds in the time of the pandemic in Poland. The article is a response to the need to understand the processes occurring in the Polish educational system under the influence of crisis events related to the pandemic. The text may prove valuable for educating future generations of teachers in the field of e-learning and increasing the effectiveness of training activities aimed at strengthening the digital competence of current teachers.

## Introduction

The COVID-19 pandemic took public services around the world by surprise. The phenomenon erupted suddenly and caused repercussions in almost every area of human life. It is now difficult to identify areas that have not been altered directly or indirectly by the global pandemic. The phenomenon has spread all over the world and has not spared the education sector either. Teachers, students, parents and education policy makers were taken by surprise by the sudden changes, the closure of schools, and the need for isolation and social distancing. These last aspects ushered in the appearance of solutions by which to communicate quickly and effectively. The rapid transformation of the educational system from the offline to the online space was seemingly easy to implement, as e-learning and blended learning were both solutions that had been under development for more than two decades in Poland. With the spread of broadband Internet in Poland, online learning gained great popularity mainly through self-education, and participation in courses organised by commercial companies, universities, and training companies. However, until now, e-learning had never been implemented completely in such a short period of time and with respect to formal (compulsory) education.

From the very beginning, this phenomenon has caused many challenges and problems, and the heightening of emotions among stakeholders focused on school facilities and non-formal education. The pandemic has become a process that has tested the level of digital maturity in individual (teachers, students, parents) and institutional (schools, educational institutions' supervisory bodies) dimensions. Taking into account the development of media pedagogy, the saturation of schools and users with new media, the implementation of long-term programmes aimed at the digitisation of education (Singh & Miah, 2020), it was this time of pandemic that provided an opportunity to quickly transform crisis e-learning into e-learning implemented in accordance with methodological rules.

According to researchers in the Canadian eLearning Network (Barbour et al., 2020), crisis e-learning can be characterised by several phases. The first phase involves the rapid transformation of the analogue environment into the digital using popular applications. Phase 2 relates to the use of the institution's resources in securing the necessary ICT equipment, allowing classes to be delivered without interruption. In addition, consideration is given in this phase to the needs of students with special educational needs. In phase 3, educational institutions are expected to provide full support to both learners and teachers. The final phase sees the emergence of full digital maturity, which means having adequate hardware resources enabling methodically implemented e-learning through the use of dedicated (as opposed to ad hoc) IT systems, closely linked to the stage of education and the learners' needs. This phase should significantly differentiate the preparation of institutions as well as teachers from the pre-pandemic situation, i.e. March 2020 (Barbour et al., 2020). The last phase closes the phase of crisis e-learning, leading stakeholders to have a high level of digital competence (both students and teachers) and adequate hardware resources, as well as efficient e-learning platforms. It is a phase that appears as an educational ideal that draws to a close the period of crisis e-learning.

This article is designed to explore the problematic situations and challenges related to the implementation of e-learning from the teachers’ perspective. This is an interesting group, one which has borne the greatest cost associated with the intensive transposition of didactics from the offline space to cyberspace. This text is an attempt to understand teachers' micro-worlds from March 2020, the period when all schools were forced to move their activities completely into the online space. Taking into account the conditions and constant features of crisis e-learning, the data collected may prove to be particularly valuable for neighbouring countries as well as for those involved in comparative pedagogical studies. The article is a unique study based on the concept of crisis e-learning and the analysis of educators' statements posted in specialized discussion groups.

## Theoretical framework and research overview

When beginning to consider crisis e-learning, it is important to start with a definition of this non-standard form of distance learning that uses information and communication technologies (ICT). By placing crisis e-learning against the background of full e-learning, which itself is implemented in accordance with methodological rules, allows us to understand the process in which the people involved in education have been participating since the beginning of the outbreak of the pandemic. Crisis e-learning is above all a process forced by external circumstances. It is not an activity that was planned in advance, which would thus have had a properly allocated budget and staff. Crisis e-learning is characterised by a non-standard speed of implementation, relying on free or readily-available solutions. The methods and forms of work change with the experience of the instructor. Monitoring the effects of training is not planned in advance and takes place on an ongoing basis through trial and error, as well as through the development of one’s own effective solutions. The forms of transmission are adapted to the possibilities of free tools and are verified concurrently with the teaching itself. Building a picture of the effectiveness of crisis e-learning is carried out on the basis of one’s own experiences and the results of the effects, changing alongside the teacher’s acquisition of proficiency in the use of online platforms and tools. Technical assistance is kept to a minimum in this type of learning. The learning content is selected and modified on an ongoing basis. The teacher is not an expert in e-learning. The differences between full e-learning and crisis e-learning are presented in Table 1.

**Table 1:** Full e-learning versus crisis e-learning

Features	Methodically implemented e-learning	Crisis e-learning
Participation costs	Minimal logistic costs	Reduced to zero due to the financial resources of students and teachers
Implementation costs	High at the beginning related to the launch of the platform	Reduced to a minimum thanks to the prevalence of free tools
Process	Possibility of high individualisation, possibility to influence the time and place of learning	Individualisation limited, place and time of learning conditioned by epidemiological situation and teacher availability
Group work	Utilising the diverse capabilities of the platforms to ensure interaction between users	Attempts to use different tools (usually well-known, recommended or free of charge) to enable group work for selected topics
Knowledge check	Automation of checking of learning outcomes The number of participants does not affect the planned knowledge testing process	Evaluation of learning outcomes on an ongoing basis with the design of new solutions to minimise cheating
Forms of communication	Using the full capabilities of dedicated platforms. Designed to be varied and to activate learners	Attempts to transpose analogue forms, methods and means into the digital environment using a variety of tools. Searching for optimal solutions
Feedback on the quality of e-learning	Use of standardised tools covering course objectives	Based on participant observation, self-reflection by the trainers, problem solving situations
Technical assistance	Provided for both instructors and learners	Low level. Peer support and self-learning occur
Development of the material	One-time preparation of material to be used in subsequent editions of the course	Development of learning materials on an ongoing basis, ongoing validation of digital content and learning resources
Tutors	Expert in e-learning, with knowledge of methodology	A person learning the principles of e-learning methodology, based on his/her own experience and drawing on the experience of other teachers in the vicinity
Source	Konieczek, 2017	Own elaboration

Crisis e-learning was implemented in many cases without adequate teacher preparation. Educators have been, as it were, forced to quickly transfer methods, content, forms, and didactic means over to digital communication environments without a basis in e-learning education methodology (Patwardhan et al., 2020). The implementation of ICT-based solutions advocated for many years has become a forced fact. Regardless of the educational stage, as of March 2020, digital synchronous and asynchronous communication channels and e-learning platforms began to be implemented in many countries as a substitute for traditional education (Spertino et al., 2020). Such a situation was the fulfilment of many idealistic assumptions that had already emerged more than two decades back. Namely, among researchers and selected groups of educators, e-learning appeared to be an effective and efficient tool that could replace traditional—analogue classes conducted in school buildings. These techno-optimists or techno-pessimists were able to revise their own views within the framework of crisis e-learning, which showed the strengths and weaknesses of the application of ICT in education (Tomczyk et al., 2017; Abdel-Hameed, 2020). Taking into account the specific features of the e-learning activities presented in Table 1, the concept of emergency e-learning will be associated with crisis e-learning, where actions are taken under the influence of the specific circumstances. Such a non-standard situation forces the transition to emergency e-learning through the use of methodological solutions and tools known from the world of e-learning as implemented in the pre-pandemic era. Emergency e-learning is related to the crisis, so the words will be used synonymously in this text.

Emergency e-learning took many by surprise, in particular those communities and groups with fewer financial resources and weaker Internet access infrastructure. Emergency e-learning also highlighted class differences, where families with high financial resources were better able to equip their own home environments with adequate ICT equipment for effective learning. Crisis e-learning was a kind of test of the capacity and flexibility of education systems in a broader socio-economic perspective (Murphy, 2021). The type of e-learning discussed also had a negative impact on the psychosocial functioning of dysfunctional children and young people. Different degrees of disability increased the number of technological challenges on the part of teachers as well as students and parents (Hutchison, 2021; Plichta, 2017; Cataudella et al., 2020). The processes discussed are not limited to one country or region, but appear to have a global character (Osman, 2020).

A completely separate and particularly relevant issue beyond the features of crisis e-learning implementation is the preparation of teachers to quickly transfer activities from the offline world to cyberspace (Alqabbani et al., 2020). A number of researchers clearly indicate that the vast majority of teachers were not prepared to implement e-learning at such short notice and with limited hardware resources (Müller et al., 2021). Based on an in-depth analysis of the available data on crisis e-learning, Bond (2020) noted that the full and successful transposition of crisis e-learning into methodical and therefore correctly implemented e-learning requires consideration of the following factors: ICT skills & knowledge; support; feedback; presence; professional development; teacher well-being; use of technology; prior ICT experience; professional networks; self-efficacy; technology acceptance; time invested; access to technology; content expertise; motivation. Analyses conducted by Bond (2020) show the multidimensionality of factors affecting e-learning. Meeting all the criteria for effective education, on the part of the student, parent, and teacher, is not an easy task and the ideal state is rarely achievable. These problematic situations were mainly solved by teachers, i.e. stakeholders who were responsible for the effectiveness of educational activities mediated by digital media. These unsupported activities, however, were subject to constant intentional or involuntary scrutiny by students, parents, other teachers, or school supervisory systems. Despite the existence of crisis e-learning, the assumed educational goals included in official curricula were still expected to be met.

## Conditions for emergency (crisis) e-learning in Poland

The decision to close the schools in Poland was made on 11 March 2020. Two weeks later, a group of scientists and didactics representing various academic and training centres dealing with teaching and development issued a pioneering publication (Pyżalski, 2020) in which, on the basis of short albeit intensive observations, discussions, and participatory research, a discussion opened on issues related to crisis e-learning. The researchers in the study posed a number of questions about the psychological situation of children and young people whose natural socialisation, development, and educational environment had been reduced to digital communicators. The researchers also sought to present appropriate methodology to be used with students in the COVID crisis. The researchers also presented the neurobiological basis of remote education. In most cases, in the initial stage of the pandemic teachers sought technical support. However, the authors of this publication devoted less space to this topic, highlighting other topics, such as setting priorities in remote education, and ways of building relationships in cyberspace. This is an important idea which guided the discussion on crisis e-learning in Poland. Digital education was not reduced by the experts to solely the effective transmission of knowledge and information. Nevertheless, in the aforementioned publication, the experts offered teachers different models for how to work with e-learning and strategies for distance learning, and presented an overview of applications supporting teachers of different types of schools and universities; they also proposed ways of developing creativity through the use of new media, and showed possible means of assessing and organising their own work. Taking into account the needs signalled by teaching staff, important threads emerged that related to the care of teachers' mental well-being. Fewer than two weeks separated the offline teaching experience and the start of online teaching, and this generated a number of challenges that went beyond the issues of digital competence, digital exclusion, or the choice of a stable and problem-free digital environment. The aforementioned publication, released just days after the closure of the schools, highlighted how many challenges teachers would face in emergency e-learning.

In order to understand the phenomenon of crisis e-learning in Poland, it is helpful to refer to some interesting results, showing a broader picture of the resources with which teachers began to perform the massive and swift digitalisation of Polish education in the time of the pandemic. Research conducted by the Digital Centre (*Centrum Cyfrowe* NGO) in April 2020 (Buchner et al., 2020) on a sample of less than a thousand respondents highlighted some interesting facts. The vast majority of Polish teachers had no experience with e-learning. Only 15% had conducted classes in this way before the outbreak of the pandemic. About half of the respondents reported encountering problems with using digital tools in education. About one third of the teachers declared that they had encountered digitally excluded pupils during the pandemic. The teachers also declared that they felt overloaded with responsibilities, while e-learning resulted in them having to perform many more activities than in the pre-pandemic period. The need to enhance the psychological and physical well-being of students and teachers due to the overload of e-learning activities also emerged from the research. (Buchner et al., 2020). In turn, the results of the research gathered by a team representing the Polish Society for Media Education (PTEM) in 34 Polish schools brought the following conclusions. Digital exclusion is not a theoretical construct (Tomczyk et al., 2019), as many Polish students "disappeared" from the educational system. This was caused by the lack of the necessary equipment to participate in remote classes. About 25 per cent of parents and one third of teachers purchased new IT equipment due to the pandemic (mainly computers, cameras, and microphones). At the end of the summer term, i.e. around July, nine out of ten teachers were positive about their own digital competences, but half of them said they needed continuous support to improve their software and hardware skills. As in the case of the previously mentioned studies, it was noted that students, teachers, and parents felt overloaded with responsibilities resulting from crisis e-learning (Ptaszek et al., 2020). The synthetically presented results were the first attempts to holistically understand the determinants of crisis e-learning in Poland. In analysing the available data (mainly quantitative) it seemed justified to undertake further in-depth (vertical) research on the determinants of crisis e-learning, and the remainder of this article will report the results of that research.

## Research methodology

### Objective and subject matter

The aim of the research was to show the characteristics of crisis e-learning in Poland from the perspective of the teachers' experiences. The research objective resulted from the situation of intensive transformation of the learning space from the analogue to the digital environment. The aim of the research covered the time when the Polish educational system was undergoing continual changes with the development of the pandemic situation. The subject of the research was provided by the statements of teachers representing formal education. The object of the research included online data posted on one of the most popular social networking sites in Poland, which were aggregated in the form of a closed group addressed to specialists in the field of education. The aim was therefore related to obtaining answers to one principle questions (Babbie, 2004; Szpunar, 2010): What types of challenges do teachers face during crisis e-learning?

The data collected allows the generalisation of the research results not only for Poland, but also for neighbouring countries, due in part to the constant features of crisis e-learning and the cultural similarities between the countries of the region. The research may also prove valuable for those conducting pedagogical comparative studies.

### Procedure and research paradigm

The research included statements made by teachers in a closed group on the social networking site Facebook. The choice of the group was intentional and was dictated by the number of members and the topics discussed. Firstly, it is the most numerous group devoted to education in Poland (over four thousand members). Secondly, the group is moderated by specialists in the field of pedagogy, so the content is focused on educational topics. Thirdly, since the outbreak of the pandemic in Poland, the analyzed group of teachers have intensified their exchange of information, experiences, tips, and case descriptions related to crisis e-learning. The authors of the study are active members of this group. Their participation in the group was accepted before the pandemic period and was in no way related to the purpose of this study.

**Fig. 1:**
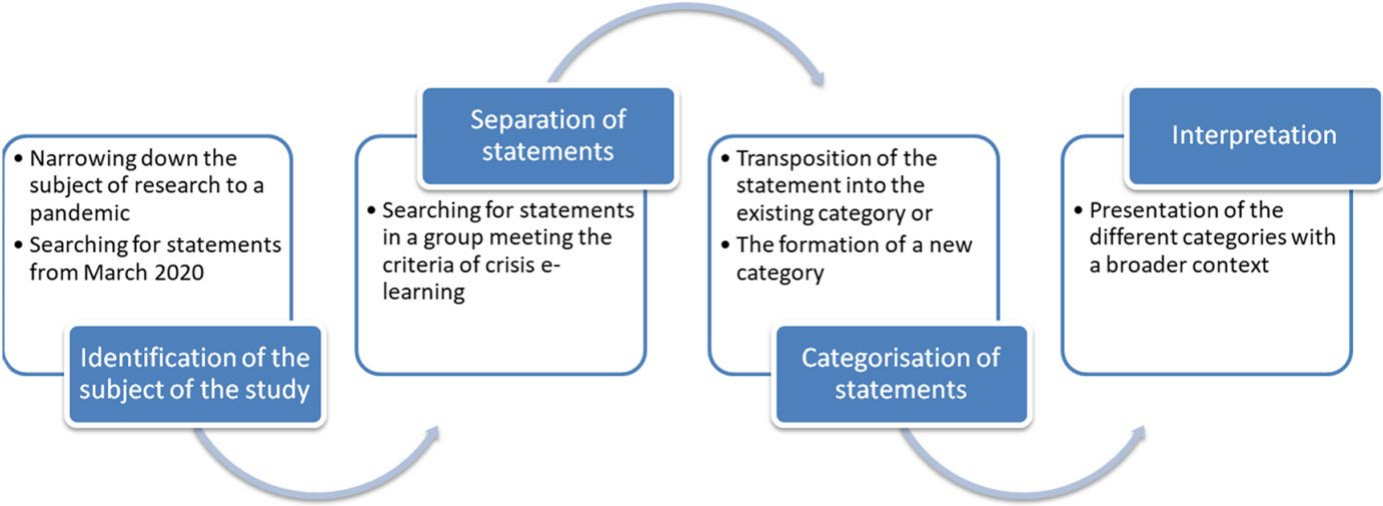
Research procedure

The research procedure consisted of the following main stages: identification of the object of research in the period March 2020—December 2020 in the Facebook group, extraction of statements related to crisis e-learning, categorization of statements, analysis of statements, and phenomenological interpretation to show the contexts related to the Polish educational system. The scheme of the research procedure is presented in Fig. 1.

Between March 2020 and December 2020, several thousand posts were analysed during the document analysis, these all being statements made by forum participants. Each post was identified in relation to the research objective and then categorised (under the condition that it is related to crisis e-learning). Statements with similar meaning were assigned to one category. The emergence of new categories resulted from the lack of a thematic link between a given statement and a previously occurring category. Repeated statements were not included in a given category due to the saturation of a given area. Thus, in the presentation of the research results, similar statements by teachers justifying the same category were not repeated also due to consideration of the length of the research report.

The applied research procedure is very often used to collect unique data on a situation which has not been sufficiently researched using "analogue" research methods, techniques, and tools (e.g. survey questionnaires, face-to-face interviews, or focus group interviews). The chosen procedure allowed, in a short time, to obtain data (teachers' statements) which would have been difficult to collect in the course of an analogue social research procedure due to limitations resulting from the epidemiological restrictions in Poland. The analysis of the teachers' statements was characterised by: (a) faster collection of data on crisis e-learning due to not needing to spend time establishing contact with the teachers, (b) high quality of data collection (statements consistent with the aim of the research presented by specialists and practitioners), (c) timeliness of data (the analysis also included the most recent categories of statements related to the current challenges of crisis e-learning), d) high flexibility of access to the data (the discussion group was freely available to those who met the access criterion), e) reaching a narrow group, i.e. teachers with experience in the field of crisis e-learning challenges and who were sharing opinions, implementations, and looking for support. The described research procedure is in line with the assumptions of new media mediated research (Jemielniak, 2019; Szpunar, 2010).

### Sample selection

The research sample was selected in a non-random way. Given the subject of the research, i.e. teachers' statements on a social networking site, it is impossible to describe intentional or stratified sampling in the case of this text. The nature of the research, i.e. the phenomenological presentation of emergency e-learning, led to each of the statements posted in the discussion group in the period March 2020 to December 2020 being analysed. The statements were only marked by gender: K—female, M—male. It is not possible to provide any additional data (e.g. length of service, place of work, place of residence) due to the camouflaging of this information in the user profiles and also due to research ethics. This is a typical research activity related to sample selection in the framework of internet content analysis. The data collected does not allow for the same kind of generalisation as quantitative diagnostic research.

### Research ethics

The research was conducted in accordance with the principles of social research ethics in the field of pedagogy. In the analysis of the statements, the personal data (e.g. name and surname, or pseudonym) of the persons giving the statements in this closed group were kept secret to maintain the anonymity of the individuals concerned. The methodology used was related to the translation of the original statements into English, thus preventing the identification of personal data. Furthermore, the group in which the statements were analysed is a closed space intended only for education professionals, so the statements presented are not indexed by external search engines, making it impossible to identify teachers. The research thus uses a double system for camouflaging personal data.

## Results

**Fig. 2:**
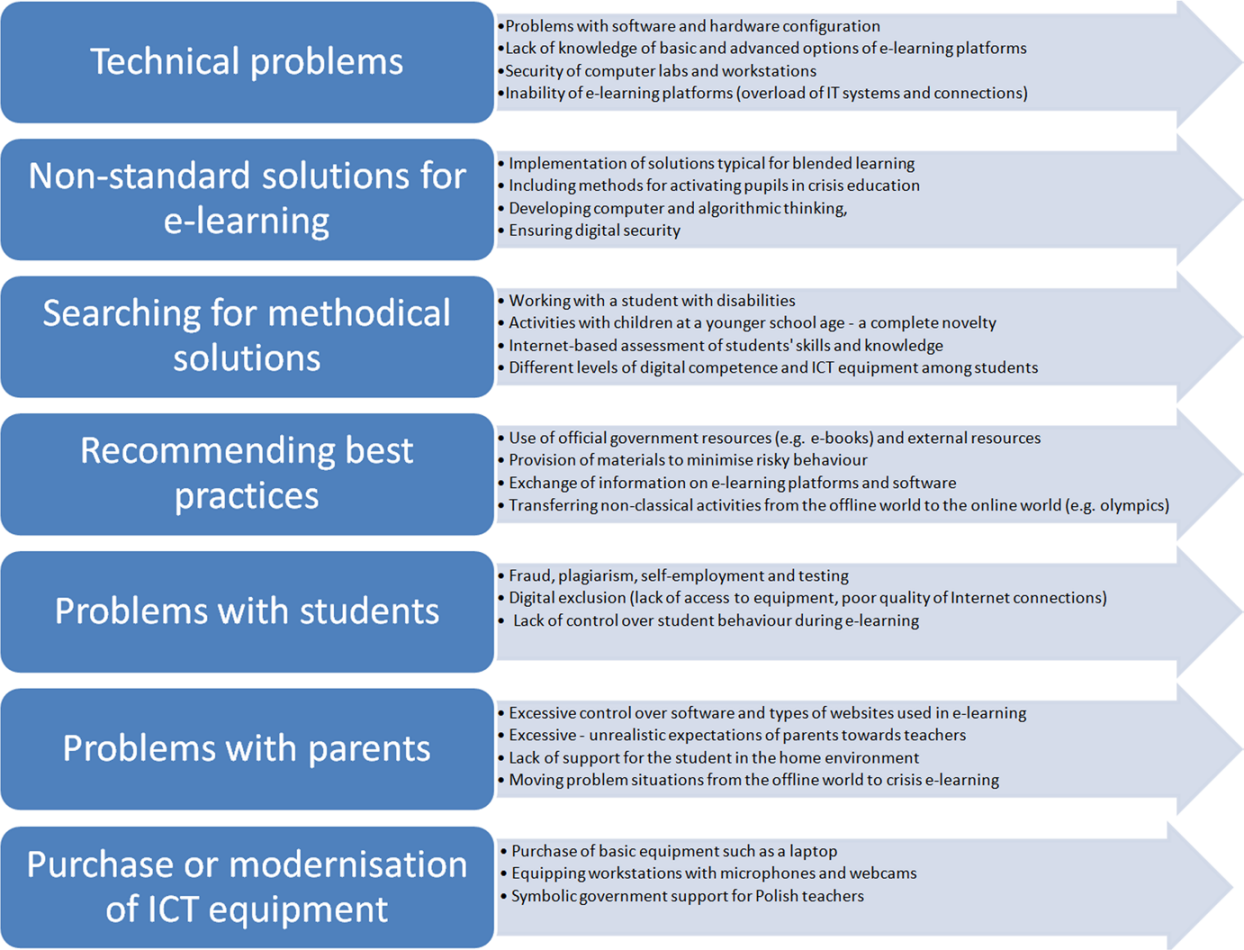
Seven categories related to crisis e-learning from the teachers' perspective

The analysis of the teachers' statements made it possible to distinguish seven categories of statements. Each refers to a different area of crisis e-learning. The first is related to technical problems that emerged during the transformation of online didactics into the digital space. The second category resulted from the use of non-standard solutions related to e-learning, which were dictated by official curricula or the development of students' interests, or the preservation of digital security. The third category referred to the search for optimal methodological solutions based on ICT and serving to improve the quality of education. The fourth category concerned the transfer of knowledge about proven solutions on the border line between ICT and education. The fifth category identified in the course of the analysis was narrowed down to typical challenges and problems occurring in the relationship between the student and the teacher. The penultimate group of data is related to problematic situations occurring between a parent and the teacher. The last group of data is limited to the modernisation and retrofitting of teachers with adequate equipment and instrumentation enabling effective communication with students. Each category is discussed in the sections that follow. A general summary of the categories together with an exemplification of the factors defining the seven categories is presented in Fig. 2.

### Resolving technical problems

The rapid transformation of the learning space generated many technical problems in the first place. Considering the data on experiences with e-learning, digital didactics, and the level of digital competence of teachers, as well as their knowledge of digital didactics, teachers especially in the initial phase of crisis e-learning declared a lot of concerns and problems related to technical aspects. It was the rapid transformation that forced the launching of solutions based on synchronous and asynchronous communication, which posed a challenge relating mainly to the handling of new software, websites, and hardware. In this respect, the respondents sought support from more experienced teachers. The range of problems associated with operating the programs varied greatly, as evidenced by the teachers' statements.

For example, there was a group of teachers who were unable to run basic applications for communicating with students. For most teachers, initiating meetings, scheduling classes, and correctly joining a class group were major challenges. This was due both to the incompatibility of the selected websites and e-learning environments in terms of hardware, as well as the teachers' narrowed digital skills. One teacher describes his problem as follows:

Maybe someone can help me here? For the past two days I have not been able to join a meeting, in any way either through the calendar or the team. When I try it says there was a problem, try again in a few minutes. K

Another group of problems occurs just after the launch of e-learning environments. These challenges are related to the correct configuration of basic student privileges. For teachers without experience in conducting e-learning classes, giving privileges to students was a problematic issue. There were many difficulties related to the learners disrupting the classes by triggering unwanted actions. Such a state of affairs not only prevented the realization of the teaching objectives, but above all forced the teachers to look for advanced options in the e-learning environments that would allow for more control over the learners' behaviour.

During online lessons in MS Teams there is a problem—students mute each other and even manage to mute the teacher. K

Communication is an elementary activity in both "analogue" and digital didactics. Efficient communication mediated by digital media provides an opportunity to achieve the desired teaching goals. For digital learning, audio and visual channels need to be configured appropriately. Selected teachers implementing specialised classes, which go beyond the administration methods (e.g., lecture, reading) seek solutions to combine different sources of knowledge transfer. Teachers beginning to explore e-learning platforms are also looking for options to improve mass communication with students. Two teachers describe the problem as follows:

Is it possible to send the same message via chat Teams only to a few selected students in a group (not to all)? I have not found such option where I can select students to whom I want to send message via chat. M

I share the screen on MS Teams and the camera image at the same time. Students say they have to switch between one and the other because the camera image cannot be enlarged (there is a thumbnail). M

In the early stages of crisis e-learning, teachers were forced to move between different versions of the software. This often generated technical problems with vision or sound. These were some of the most common problems that prevented efficient teaching.

Hi, I have a question about Google Meet, is it possible to share a presentation or whiteboard and at the same time see the students as in Zoom. So far we have been using Zoom, now we are moving to Gsuite. There will be lessons in Google Meet. K

Hiya, can you please advise how to solve a problem with a bluetooth speakerphone (headphones with microphone). It does not want to work i.e. either the headphones or the microphone work M

Teachers familiarising themselves with the possibilities of the e-learning platforms pay attention to problematic situations that appear unintentionally and are difficult to resolve. The lack of experience among educators in removing non-standard options that hinder lesson delivery forces them to seek support from those more competent in the field. Therefore, in the analyzed discussion group there are many requests related to the search for solutions related to the operation of e-learning environments. In the vast majority of cases, teachers seeking support receive very precise answers allowing them to solve the problem.

I accidentally deleted my group calendars from classroom. How can I recover them or create new ones. I don't know what address to enter when creating a new one…I think I've tried everything. K

With technical support also comes the search for solutions to improve digital security. However, this generates many technical-ethical-legal problems. Some of the solutions are consulted among other teachers. In particular, teachers are interested in the issue of protecting computers that are owned by the school and have been loaned for the duration of the pandemic to digitally excluded students (those without basic equipment).

Can a monitoring program be installed on a school computer loaned to a student? This is one idea I have read about. I don't mean Microsoft solutions. I mean external tools. M

A separate group of technical problems covered the mixed mode and different stages of crisis teaching. With the start of the new school year 2020/2021, some pupils returned to school for a short period (traditional teaching). This necessitated teachers to prepare safe workstations, thus minimising exposure to virus infection. In most schools, during the summer holiday period (July—August 2020), standards for residential teaching for the new school year 2020/2021 and for the mixed mode were developed. The latter mode also took into account the situation when students, due to a lack of equipment, absence from online classes, or Internet problems, participated in crisis e-learning on the school premises e.g. the school lab.

Does anyone in the group know if it is possible or have experience with ozone treatment in a computer lab? M

Crisis e-learning has forced a change of mode on all teachers. It is clear that teachers have reformulated the methodology of preparing materials to be compatible with remote learning, which in most cases (as noted in the introduction—the theoretical framework), has forced an increase in the number of hours devoted to preparing digital materials. However, there is another thread to the same process. In Polish schools, the first support in crisis e-learning was provided by teachers of computer science and computer classes. As digital competence classes are obligatory in Poland, every school has a specialist on the borderline between computer science and pedagogy. These persons have to a large extent taken over the responsibility for the digitisation of their own schools. Very often such activities were conducted without much financial support and generated dissatisfaction in this group. These issues were expressed by the teachers as follows:

Do any of you have an extra charge for the administration of teams and librus? I ask because I have been working much more than 40 h a week for the last while. Higher incentive, extra full-time/half-technical hours for administrative support? K

I have been given the task of finding the best tool for online (real-time) lessons. The principal gives free rein, the only requirement is that the tool should support up to 300 students. Could you suggest me something? K

Technical problems include not only the above-mentioned emergencies and challenges related to the appropriate configuration of hardware and software, but also the inefficiency of selected and popular IT systems. In the initial period of crisis teaching, the overloading of popular servers supporting access to educational data and enabling online transmission was noticeable. The pandemic proved to be a situation that tested the performance of the most popular solutions used in education and beyond.

I can hardly see this remote learning. Already now the vulcan log crashes frequently, padlet is overloaded at 17, even Facebook is out of breath from time to time, youtube and netflix cut the quality in order not to overload the network. M

The technical problems presented took different forms. As time passed and more experience was acquired, teachers were able to solve selected groups of problems. Natural familiarisation with basic and more advanced options, peer counselling, self-education, and seeking specialist support in the form of courses minimised the sense of technical helplessness. Nevertheless, the group of technical problems was one of the most noticeable categories emerging from the teachers' responses and questions.

### Searching for information on non-standard solutions for e-learning

From March 2020 to December 2020, school activities were mostly conducted remotely. The theoretical part briefly presents the first results of the research summarising the technical and methodological issues from the perspective of quantitative research (the scale of the phenomena). However, this research does not capture the intermediate modes, i.e. the situations that took place between September 2020 and mid-October 2020, during which time the classes were conducted in a stationary manner in schools. There was during this time a group of students who stayed at home due to the epidemiological risk – they had either tested positive for the virus or were showing symptoms of viral infection. Teachers searched for a period of one and a half months for solutions to combine the stationary mode with an online mode dedicated to pupils in quarantine at home.

We have such a situation: a child in quarantine, but his class is studying normally at school. PPIS confirms that the child's quarantine will be long—about a month…. We want the child to use the lessons at school and participate remotely. M

Non-standard solutions for e-learning usually concerned activities that went beyond the classical activities assigned to teaching and learning. Teachers wishing to increase the attractiveness of classes sought information on non-classical forms of communication with students in the discussion group. Despite the pandemonium, the teachers also searched for organisational solutions that would allow them to hold thematic competitions. The search for non-standard solutions required transposing experiences from the analogue world into cyberspace, while taking into account individual and environmental conditions.

You know, there used to be (and probably is) a portal where I could type in a question and the participants their answers and from those answers a word cloud would build up… I can't remember the name… M

I am looking for some guidance, advice. A group of pupils from my school, each with their own individual voice, are to record themselves singing the school anthem. I am supposed to put all the recordings together to create an "online choir". The end result should be that there are many windows with the students' recordings on the screen at the same time. K

Non-standard ideas also concerned activities developing programming thinking, commutation, and strengthening technical skills in robotics. Such an area of research is not accidental, but rather results from the reform of Polish education oriented at developing IT skills through solutions known and liked by students, which are then used during IT lessons. One teacher illustrates the following situation:

Do you guys have any ideas on how to run robotics online? I have Robobloq and Arduino a couple of kits. I was initially thinking of having them prepare the finished algorithms themselves in the robot software, email them, and I upload and show how it works. However, it seems to me that there will be a lot of confusion about this. Do you have any ideas on how this could be done? M

Teachers are also aware of maintaining digital security in a crisis teaching situation caused by the global pandemic. The transformation of activities from the offline world to cyberspace increases the time spent online. This, in turn, means that the processes associated with the use of e-learning platforms and the use of digital media, among other things, are characterised by an increased risk of exposure to undesirable situations and behaviour. Monitoring and securing both e-learning platforms and individual workstations is treated by many teachers as a priority. Such an approach is also enforced by the current European Union regulations guaranteeing the protection of personal data.

What security problems do you have/have you experienced with your private or work Moodle platforms hosted on hosts or servers? Have you had issues such as SQL injection or other issues that could be related to unauthorised access to your courses? K

I have a question. Is there a program that allows you to see on the parent's computer what windows and how the child is working on the computer next door in the room? K

Through the intensive transformation of the learning space, teachers have also discovered applications that are very often used by young people. A group of educators, involved in an externally induced life long learning process, attempted to understand and use the apps and websites popular among children and young people.

Do you use Discord? Is it worth getting into? What are the advantages/disadvantages? M

The rapid and at times chaotic transformation of communication and learning spaces has necessitated the exploration of new ICT-based methods, applications, and websites. Undoubtedly, crisis e-learning forced the updating of digital competences and going beyond the typical use of new media known from the pre-pandemic stage.

### Search for methodological solutions

In the first phase of crisis e-learning, teachers reflected on the possibility of engaging students in regular classes conducted online. According to research conducted in Poland (Buchner et al., 2020), the vast majority of teachers had no previous experience in the implementation of the core curriculum mediated entirely by new media. This condition raised many concerns from the very beginning.

I wonder how many pupils will want to spend time at computers during this break from school for educational purposes? I guess support from parents will be needed. K

The transformation of teaching methods, forms, and means during the pandemic did not bypass teachers working with students with special educational needs. Special educators are a unique group that has not been adequately prepared for remote activities with students with deficits. Such activities are made much more complex and complicated due to motor, intellectual, and sensory limitations that affect the process of education mediated by new media. The topic of teachers who are special educators could be a separate study due to the complexity of the methodology of working with disabled students using cyberspace resources.

A grade 5 pupil with Asperger's. I have had one-to-one teaching with him so far. Now we are on remote and I don't know how to bite the topic. The pupil has impaired fine motor skills in his hand. He has a hard time using the mouse and doesn't like or want to learn new technologies. The only thing he wanted to do was paint drawings. During individual lessons I gently tried to sneak something in, but without success. Now I have no idea how to work with him. K

The biggest challenge proved to be the didactic work in the e-learning mode with the youngest students. It was education aimed at children at the first stage of education that proved to be a complex didactical challenge. This was due to the fact that it was a complete novelty for early childhood education teachers. While teachers at later stages of education possessed some general knowledge of e-learning, teachers of the youngest grades were characterised by a very high level of anxiety about the effectiveness of their lessons.

It has happened. Children 1–3 have online classes. What ideas do you have for working remotely with such little ones? Today I had my first class 3, it turns out that half have computers, the other half tablets and individual cases are working from their phones.

Tomorrow I have my first ever remote computer science lesson with class 1. Do you have any ideas or suggestions on what to do with these young children? K

The effectiveness of teaching is very often measured by test results. Testing knowledge is not only a motivating factor for learning, but also a kind of 'litmus test' to check the effectiveness of the activities carried out in cyberspace. Many of the teachers interviewed assumed that online testing would be one of the main ways to test students' knowledge. Teachers shared with each other on an ongoing basis best practices to avoid student cheating and to build ongoing knowledge of the achievement of learning objectives.

Is there any point in taking tests online? How do they work? K

I do not think we have a choice. Testportal severely limits downloading. That leaves the phone, but time is of the essence so the results are fairly reliable K

I do tests on Moodle—I create a database of questions and the system randomly selects questions, in addition the answers are mixed. One question is displayed on the page and there is no way to go back to the previous ones. M

The digital competence of students also presents a challenge. Although today's students are a generation of so-called digital natives, supposedly capable of handling new media without any problems, teachers point to the fact that this group is not homogeneous due to different levels of knowledge and skills in handling new media. Teachers have knowledge and skills concerning differentiation of methods and forms of education in the traditional—analogue educational process; however, transferring certain solutions from the offline sphere into the online space becomes a methodological challenge due to the different levels of digital competence of the students.

Hello, I'm looking for ideas for a cool computer science lesson for class 5. The problem is that there is a very big difference in the students' skills in the class. Half of the class can barely operate a computer, the other half are at a much higher level than the curriculum stipulates. It is only in this one class that I have this problem. K

Teaching narrowly specialized subjects such as computer science becomes problematic when students use different hardware resources. In the school laboratory, the implementation of computer science classes follows a standardized scheme, which results from computer laboratories having the same hardware and software at all workstations. In crisis e-learning, the situation related to the diversity of hardware, software, and licenses for the use of software raises many concerns, as evidenced by the following statements.

I joined the group because I am running out of ideas. Secondary school, 2nd grade. Students at home do not have Office, A1 school licence is limited. There is no Access and no mail merge in Word.. K

In my opinion (supported by my own observations during almost 30 years of teaching computer science) it is a very harmful practice to teach only one programme. For example, I have always done a lesson on creating mathematical formulas (chemical, physical) in LibreOffice (previously in OpenOffice) and another lesson with the same formulas in MS Word editor. Otherwise it will be like teaching Photoshop and Corel on courses for the unemployed. Sure they won't buy it for themselves, and they don't know the other software. They will use cracked versions—which means thievery. M

Crisis e-learning raises many questions about the components of an effective e-learning methodology. These include issues related to software, hardware, level of digital competence, legal aspects, and the specifics of the target group (e.g. the developmental period of the students), or the type of subject being taught. The indicated statements unequivocally prove the impossibility of the easy unification of the learning and teaching process. This is a phenomenon which also occurs in classical—stationary teaching; however, within e-learning it gains particular significance due to the multidimensional challenges seen in the statements made thus far.

### Recommending best practice

The analysis of the teachers' statements allowed a further category to be identified. These are statements recommending websites and software that increase the effectiveness of teaching and learning. Teachers' statements in this area constitute a repository of tested, categorised solutions that can be used by other educators. In the situation of crisis e-learning, such statements were very popular among educators looking for effective software and websites, as evidenced by the feedback comments or the number of likes on a given post. Many of the messages were about solutions aimed at children of younger school ages. In the vast majority of cases, the posts referred to other sites (outside the social network) run by methodologists, new technology enthusiasts or hobby teachers sharing information on the implementation of pedagogical innovations.

I recommend Christmas fun for recent computer classes 1–3 (and beyond), especially those on coding. K

How to organise lessons in classes 1–3 in Microsoft Teams. How to control the classroom and how to set up so that children do not sit for long periods at the computer. There is more information about this here. M

For teachers, lesson delivery starts earlier than for students. The teacher prepares the conception of a new lesson conducted remotely in the preparation phase, thus much earlier. At this stage, he/she searches for teaching materials, prepares an official or working lesson plan, develops exercises, and searches for materials in available official resources (e.g. e-books or tutorials). Proven solutions are very often advertised by teachers who have made didactic implementations in their own school.

How to present a quick and interesting lesson in Microsoft Teams Microsoft Sway—A cool way to give presentations as a web page. Let me know if you find it useful? M

In addition to didactic solutions, there is also information related to upbringing in cyberspace and solving e-threats. Teachers on the platform share examples where students have prepared solutions to minimise the effects of cyberbullying directed at teachers and other students. In their statements, the teachers also exchange links to materials from where teaching materials for minimising undesirable behaviour among students can be downloaded within the framework of crisis e-learning.

In our town, one high school "went wild". However, the culprits were found and had to show off the other way as punishment. The class … apologises to all the teachers who were harmed by the videos they released. As part of the apology, a video has been produced which includes the apology and a brief discussion of why such things should not be done. K

With the development of the pandemic and the increasingly intensive transformation of "analogue" didactics into cyberspace, there was an increase in the intensity of exchange of opinions on the various platforms on which the teachers implemented formal (compulsory) education. In Poland, a high degree of liberality was adopted towards the choice of e-learning environment. This voluntariness resulted not so much from the independence of schools from the entities supervising them (e.g. the School Superintendent's Office or the Ministry), but from the features of crisis e-learning (e.g. no common strategy, no central methodological support). Teachers using specialist groups supported each other from the bottom up and independently, based on their own successes and failures with testing particular online platforms.

From mid-March onwards, teachers started to look for remote learning platforms. In one scenario the principal left them alone and as a result the student had to switch to a different platform every lesson and in the other it was up to the school to deploy some system. The choice was between big brands such as Zoom (but fell off its high horse at the end of March due to suspected data leaks to China and reported hacks of remote lessons), Microsoft Teams, Discord and many others. None of these platforms, however, make the shortcomings of Windows support go away. And this is where inconspicuous little programs come to the rescue, after installing which we cannot imagine life without them. M

If you're getting started with Minecraft Education Edition then I'd like to invite you to a free webinar—I'll talk about how to attach students to your world, how to create a world and what possibilities MEE offers. M

In view of the announced pandemic and the two-week break in the functioning of schools—what ideas do you have for replacing traditional education with e-learning tools? I personally bet on YouTube and transferring materials using OneDrive cloud. As for testing the students' knowledge—the quizizz.com platform. M

Recommending good practices went beyond formal education. In Polish discussion groups (not only those analysed in this paper), one can find statements encouraging teachers to participate in additional events, e.g. competitions and Olympiads. This means that despite the crisis-pandemic situation, many activities occurring before March 2020 also took place during the lock down. Teachers were able to transpose activities from the offline world into cyberspace and to share information about such activities. Many of these events were supported by commercial entities working within the framework of corporate social responsibility.

Have you heard about European Coding Week (CodeWeek)? It is Europe's biggest programming event, which aims to promote coding as a skill that develops everyone and helps understand our increasingly technology-dependent world. This year, it will run from 10 to 25 October (yes, yes, the coding 'week' lasts two weeks). M

Polish teachers in the e-learning crisis situation acted without central support. However, the group was able to implement new ICT-based solutions at that time. Positive and negative experiences were presented in discussion groups bringing together educators seeking answers to problems as well as sharing their own ideas and teaching successes.

### Problems with students

Crisis e-learning in the first instance triggered many technical problems. In subsequent stages of rapid digital transformation, challenges emerged related to the maintenance of discipline, the transparency of assessment of student performance, or the true involvement of students in educational activities. Crisis e-learning, as noted in the introduction, is characterized by different dynamics of implementing digital solutions than e-learning implemented methodically, systematically, and in the long term. One of the many problems, and which was particularly highlighted by the teachers, was the issue of the reliability of the tasks performed by students. Teachers very often had the impression that their students were not approaching the lessons in the right spirit, and that cheating was becoming increasingly widespread.

I have two pupils and I am almost certain that the work I received for assessment in their case was done by one and the same person. The works are identical (Baltie). How can I check the file data (e.g. creation time…) anything to determine if it is the same work and only the same? Students rather weak and work done very well. K

If a person writes a test on Testportal twice under different names, is it possible to prove somehow that it was the same person? For example, Basia Kowalska writes a test as Jan Malinowski, watches the tasks and then logs in under her own name. Can I somehow prove that the test was written by the same person? K

The issue of problems disrupting the learning process is strongly linked to digital exclusion. Crisis e-learning found some families unprepared for the intensive use of ICT by both adults and children at the same time. Since March 2020, the demand for ICT in home environments has increased due to the intensive transformation of many services typically found in offline spaces into their online counterparts. Many parents have been forced into remote working mode, resulting in restrictions on access to digital equipment in the home environment. Digital exclusion has primarily affected students from urban affluent families, dysfunctional backgrounds and those living in areas without access to broadband Internet. However, the lack of equipment (e.g. computers) was also present among non-functional families; however, with limited financial resources allowing the equipping of each member of the household with an independent computer with high-speed Internet access. The problem of digital exclusion was strongly emphasised by many teachers since the beginning of the pandemic.

And what to do when pupils don't have a computer, tablet or sometimes even a phone at home, or one computer for every five children. K

Teachers in many didactic and educational situations do not have a sense of influence on student behaviour, including the implementation of instructions conducive to the achievement of objectives arising from the core curriculum. Crisis teaching has generated a number of problems at the intersection of technology, didactics, and educational impact that have not previously existed.

Hi, I have this question: I want to teach kids how to use Google Drive, share work, use a text document, presentations, etc. However, one student has refused to log in to his Gmail at school. Maybe? What are the legal aspects? K

You need to talk to the parents that this is necessary for the core curriculum and that it gives them access to the material from the lesson. You can ask them to decline in writing and not to wish the student to follow the programme. K

Problems with student relationships occur in both the online and offline spaces. Technology reinforces and generates new challenges that teachers need to address in order not only to continue the teaching process, but also to demonstrate their own effectiveness and efficiency among the class team. The issues of student problems in crisis teaching should also be considered somewhat more broadly, for example in the context of cyberbullying directed at teachers. However, this issue is very broad and should be explored in a separate study.

Issues of fraud are not a new phenomenon, and occur in both online and offline spaces. Emergency e-learning has only served to highlight selected forms of behaviour that are classified as negative. Students who are deprived of the classic relationship with the teacher, who are overloaded with mediated contact, who are overloaded with educational material, react problematically to these situations. The cheating, cyberbullying, educational passivity, multitasking, and inappropriate behaviour that have all emerged during the period of online education would be an area worth considering, though as a separate article showing the dark sides of e-learning in the time of the pandemic.

### Relationship problems with parents

The description of the problems faced by children and young people in the analysed group was not as extensive as the issue of the relationship with the students’ parents. In the vast majority of cases, teachers are able to solve classic educational problems both in the online and offline world, which is due to both their accumulated professional experience and the support of other teachers or the institution of appropriate school procedures. Nevertheless, crisis e-learning has shortened the distance between teachers and the parents of students. Parents very often, especially during the first phase of crisis e-learning, participated in the classes delivered remotely. Teachers often looked for teaching materials and solutions based on services with increased interactivity using popular, freely available e-services. This situation did not always meet with the approval of parents and at the same time caused consternation among teachers. One example is described below.

I received a message with this content from one of my parents. What do you think? What can be done/responded to? <  < You are sending out materials about children's addiction to the Internet, while as a school you are contributing to it. I'm not talking about e-learning as such, I know that nobody from the school has any influence on that. However, the school does have an influence on the content channels. Teachers are constantly uploading material to YouTube. This is unacceptable to me. This is a medium available from the age of 13, which means that the school is forcing parents to break the rules and, what is worse, to give our children access to inappropriate content. After all, it is possible to create profiles for children on computers, to create a slightly safer environment for them on the Internet. But what good is such a profile if a child who wants to do homework has to be logged into an adult profile, because children's accounts do not support YouTube? K

Parental expectations are a constant in the life of the teacher, but are exacerbated by crisis e-learning as seen during the pandemic. There is a group of expectations that are difficult for school staff to meet. This is primarily due to the characteristics of mediated communication. At the same time, in the requirements of the parents, there emerges a broader context of problems concerning the style of use of new technologies by students in distance education. Both parents and teachers note that students, when multitasking, do not carry out activities that stem from their school duties; in other words, the students are often engaged in more than one activity during online lessons, and are not solely devoted to paying attention to the lesson. In the teachers' statements it is noted that the responsibility for this issue is fully ceded by selected groups of parents to the teachers, despite the fact that in reality the student is in the home environment.

You also have this in your schools: parents expect us to have an influence during distance learning so that pupils don't play games, don't look at other websites or apps, don't consult each other about tests and exams, because, as they say, they care about their future. K

Statements from different categories can overlap. One such example is the issue related to digital exclusion. The lack of access to synchronous (preferred) as well as asynchronous communication is not uncommon. Lack of access causes frustration among both parents and teachers. Educators responsible for the process of remote education emphasize that the appropriate preparation of the student for e-learning rests with the parents or legal guardians. Access to educational platforms and means of communication is implemented in many ways taking into account the financial and hardware resources of the family. However, this issue raises many emotions and disputes, as evidenced by the following examples.

A parent stated that a student (grade 7) does not have a computer or laptop at home and only uses his phone for distance learning. What about computer science can be done on the phone? K

If there is no equipment then borrow from the school or the child can come to school and use the school equipment. If he jumps out of his pyjamas a few times and gets up at 7 instead of 7.55, the equipment can be found quickly. This is how it was for us K

When discussing digital exclusion, teachers also raise arguments related to government social support for Polish families, which makes it possible to minimise this unfavourable phenomenon. Teachers are aware of the fact that the purchase of equipment enabling access to popular e-learning tools does not require technically advanced equipment, whereas social programmes existing in Poland make it possible to eliminate the problem in a very short time.

There is 500 + [a monthly stipend of 500PLN per child] and it is clearly stated to benefit children. Is it difficult to buy a laptop or a computer for, let's say, about PLN 1,000? Even a computer for 300 PLN will work. I know that money is not always easy to come by, but it may sound bad—the ones who shout the most are those who have money. M

Crisis teaching using e-learning raises many emotions. Problematic situations in family life also spill over into the relationship between parents and teachers. School staff show a double strain when parents do not provide access to basic equipment and burden teachers with their demands at the same time.

Is it within the teacher's competence to send text messages, mms with programming work because the child does not have a computer? This is what the child's father demands of me. The child has programming classes with me—class III primary school. The parents are divorced, the father took away the child's computer, "cut off" the internet. I quote the father's words: <  < How the teaching of programming is supposed to proceed you should know this and be prepared for different possibilities. K

There is also a group of parents excessively controlling the process of crisis e-learning. Situations going beyond classical solutions for analogue didactics are quickly caught by parents in this group and classified as mistakes requiring immediate and absolute correction. An example of such a situation is seen in the following statement:

"I love" parents who have made it their life's goal to control the teacher's every move. As an interesting side note, in our case a parent wrote a complaint that the lesson lasted 47 min. K

E-learning does not happen without the participation of parents. It is the parents who provide the basic learning resources to enable the child to learn. In the era of crisis e-learning, ICT is such a basic solution. However, although the COVID pandemic has occurred against the background of the intensive development of the information society, this elementary condition related to access to equipment is not always fulfilled. The responsibility of some parents to ensure access to equipment is transferred to the schools, thus raising concerns and misunderstandings between parents and teachers. Such claims and the generation of problematic situations are a noticeable phenomenon. However, this is not a situation that occurs only in crisis e-learning.

### Purchase of equipment

Another equally important category of problems raised by teachers is the issue of the selection of equipment adequate for classes conducted in the e-learning mode. Teachers who are not IT specialists very often look for information on the choice of given solutions, their affordability and applicability within the e-learning crisis. As a first step, many educators sought information on laptops. This question was related to the lack of institutional provision of support for teachers in the area of retrofitting this group with basic ICT.

I know this question has already been asked, but I can't find it. Which "recycled" laptop should I choose if I have 1500 PLN max? K

Instead of a laptop, a new desktop computer at this price with a used monitor can be bought without major problems M

Another category of questions going beyond laptops and desktops was related to microphones and webcams. Those using desktops at home that did not have this kind of additional equipment during the e-learning crisis were looking for information about devices that would enable full audiovisual communication with students. Some teachers were also dissatisfied with the quality of standard laptop equipment and therefore sought information to improve the quality of the transmission of their classes.

I need your advice on choosing headphones for online meetings via MS Teams, Zoom, Messenger. What type of over-ear headphones with a microphone will work well here? Are headphones with a headband (extended directional microphone) or a microphone on a cable a better option? Will wireless headphones with a built-in Bluetooth microphone also work here? I will be conducting online meetings in a closed room without any third parties. M

What kind of classroom camera do you recommend. So many of them. K

Polish teachers in the first phase of crisis e-learning did not receive central government support allowing them to purchase computers and additional equipment. Many of the teachers used resources located on school premises, e.g. in computer laboratories. Another group used previously purchased equipment. There was also a number of teachers who purchased equipment shortly after the lockdown was announced. In the second half of November 2020, the Polish government announced support in the form of a grant of 500 PLN (approximately 110 EUR) for the purchase of equipment for teachers. However, this funding was not sufficient to purchase a fully equipped workstation to deliver effective e-learning.

## Discussion

Seven categories of challenges and problems were extracted from the analyses of the teachers' statements. These perspectives show the most noticeable features of crisis e-learning in terms of teachers' experiences. The first category refers to technical problems related to hardware and software. In the first phase of crisis e-learning, most teachers were forced to launch e-learning solutions on their own without adequate technical support. This situation generated many problems, preventing effective education. Technical problems were conditioned by teachers' poor experience in e-learning (Pyżalski, 2020), as well as the level of digital competence of the teachers (Borthwick & Hansen, 2017; Stošić & Stošić, 2015), and also revealed the level of preparation of young teachers for their profession in the era of the information society (List, 2019). Technical issues in the first case were an automatic diagnosis of the level of digital maturity, one of the indicators of which is the ability to solve professional problems using ICT (Balaban et al., 2018; Gill & VanBoskirk, 2016). Technical problems were also linked to the category of low-quality retrofitting of institutions and teachers with equipment to implement e-learning. Although the e-learning crisis was preceded by diagnoses on the quality of ICT use in education, conclusions from earlier studies (Plebańska, 2018) and government programmes so far enacted have not brought the saturation of IT equipment in Polish education to a satisfactory level.

The second type of statements was related to the implementation of non-standard solutions in crisis e-learning. With the improvement of ICT proficiency with a particular emphasis on e-learning platforms, the demand for diversification of methods and forms of education was growing. Teachers slowly recognised the possibilities inherent in e-learning platforms and supporting software. This stage was related not only to the strengthening of the digital competence of teachers, but also resulted from the natural didactic principle of diversifying methods of working with students. E-learning in many elementary ways does not differ significantly from the assumptions of general didactics (Pyżalski, 2020), but has a much wider palette of possibilities resulting from multimedia (Plebańska, 2011). The second category of teachers' statements was strongly related to the third one, i.e. the search for proven and effective e-learning solutions. The teachers in the specialised discussion group were very willing to share information about the software they had used and rated highly. What was evident here was the diversity of topics resulting from the subjects taught and the versatility of the selected software, which could be used in various content contexts of formal education. This category testified to the increasing awareness of teachers about e-learning and their willingness to share knowledge in their professional environment. Crisis e-learning also triggered many positive consequences, including those related to self-learning (Abrossimova et al., 2020). Teachers were very willing to share their own experiences of issues that have not been sufficiently described in the literature so far, including the use of e-learning in pre-school or early childhood education and working with students with disabilities (Stoiljković, 2020).

Crisis e-learning turned out to be a phenomenon that highlighted problems relating to the relationship between the teacher and with students and parents. Mediated communication channels, a lack of empathy, the speed and chaotic transformation of learning environments, confusion, and problems in the offline world were significantly related to the emergence of problematic and conflicting situations between the teacher and the parent and student. On the one hand the teachers were overwhelmed by the amount of additional responsibilities. On the other hand they had to deal with negative feedback, so to speak, evaluating directly or indirectly the effects of their work, as well as the crisis e-learning process. Negative issues relating to the evaluation of the activities undertaken by the teachers on the part of the parents resounded very strongly in the statements made by the teachers. The teachers highlighted the issue of the lack of support from parents, the generation of conflict situations, entitlement, and unrealistic expectations that have nothing to do with crisis e-learning (Amanor-Mfoafo et al., 2020; ElSaheli-Elhage, 2020). The contributions presented strongly highlight the importance of treating education in a broader perspective. Achieving the intended learning outcomes is not a simple sum of teacher-student relationships plus activities, but should take into account the wider ecosystem contexts, including the parents (Bhamani et al., 2020).

Crisis e-learning was a phenomenon that took every country by surprise. Countries with high levels of digital literacy development, as well as extensive open educational resources, investing in teacher preparation for the use of digital media in education (Arteaga et al., 2020; Dhawan, 2020) proved to be much less vulnerable to disruptions resulting from the rapid transformation of educational systems due to COVID (Lestari et al., 2020; Rush et al., 2014). When conducting r analyses devoted to crisis e-learning, it is also necessary to ask about the level of preparation for being a learner in an environment mediated by new media (Díez-Gutiérrez & Espinoza, 2020; Fedeli, 2012). Effective learning in the era of crisis e-learning is not exclusively linked to the preparation of educational bodies and teachers, but is also linked to the ability to learn through new media in different age groups.

Crisis e-learning has triggered many changes in the Polish educational system. It is certain that it has strengthened teachers' and parents' abilities to use new media in education (Romaniuk & Łukasiewicz-Wieleba, 2020). Despite its overall negative character, the pandemic contributed to the rapid transformation of educational spaces. These transformations were at times chaotic (especially in the first phase), burdening teachers as well as students and parents with high costs. In the long term, however, this phenomenon may bring positive consequences. These include: strengthening digital competences, retrofitting ICT equipment, encouraging experimentation with educational software, and conducting in-depth analyses of the changes in the lives of modern societies through digital media (Minghat et al., 2020; Hasan & Bao, 2020). Taking into account the elements mentioned, at the same time, it is necessary to be aware that there is a group of teachers who, despite the attempts related to e-learning, have developed negative characteristics oriented towards the use of media in education due to the overwhelming number of technical, educational, and didactic problems they have faced. Crisis e-learning should therefore not be considered dualistically but should take into account broader contexts linked to the microcosms of teachers' and learners' experiences (Adarkwah, 2020; Almaiah et al., 2020).

This article provides a different perspective of the e-learning crisis. The data collected show how many problems both students and teachers face during the forced move to digitization. Of course, these qualitative data do not show the scale of the phenomenon, but they highlight problematic situations in a descriptive way. Such research reports can be particularly useful for teachers who can relate to their own problems in crisis e-learning through the statements of others from within the educational sector. The teachers' statements also reveal their own level of digital maturity as well as that of their students and the students’ parents. The text shows the current stage of development of media pedagogy and is a voice in the discussion of the global dimensions of pandemic learning, which goes beyond the full, appropriate and long-term implementation of e-learning.

## Research limitations and orientations

The research presented was not carried out in a way that allows generalisation. The statements analysed were all sourced from a closed community whose members were by definition active in cyberspace. Many teachers may have a completely different perspective on the challenges of crisis e-learning, but do not share their opinions online for various reasons (e.g. lack of knowledge about discussion groups, lack of motivation to share knowledge, or low digital competence). The perspective presented is more a presentation of a subjective microcosm than of events that have taken place in every school since the beginning of the COVID-19 epidemic. Although the number of statements analysed is significant, in the applied research model it is not possible to speak of a full saturation of the categories delineated here, since many teachers likely do not know about these online discussion groups, or if they do, refrain from participating for whatever reason. These are closed circles for those motivated to discuss digital didactics and for those looking to improve their own teaching.

In the next stage of the research, it would be useful to carry out further analyses on the perception of the changes brought about by crisis e-learning from a longer perspective. The use of qualitative methodology may be useful in revealing the mechanisms accompanying the change in the level of digital competence and the knowledge of digital didactics due to the pandemic crisis that transformed formal education. It also seems reasonable to measure the changes in attitudes towards new media through e-learning. The topic of crisis e-learning will likely be a constant point of reference for the development of media pedagogy and will provoke many discussions on preparing the educational system for the use of new media in the process of education and child development.

## Conclusion

The Polish experience should be considered in the broader perspective. The data collected provide a basis for the comparison of teaching experiences in many countries in the region given their many similarities. The research review and the statements collected so far clearly suggest that crisis e-learning is characterised by several constant global aspects, i.e. it is unplanned, causes a rapid transformation of teaching methods and forms, generates challenges and problems on the teacher-student line, and triggers a number of discussions on the effectiveness of teaching mediated by new media (Eger, 2020).

This text has sought to explore the range of problems faced by teachers in the era of the COVID pandemic. In the course of categorising the contributions it was noted that the e-learning crisis generated many different problem areas that required ongoing, often novel, solutions. Polish educators, as well as representatives of the education sector from other countries (with some exceptions – such as those countries with a high level of computerization of education) (Hu, 2017; Tomczyk & Oyelere, 2019; Tomczyk et al., 2020) were not prepared for the full transfer of educational activities into cyberspace. The crisis situation has highlighted the importance of improving digital competences, as well as knowledge and skills related to digital didactics, or e-learning. These activities serve to raise the overall level of digital maturity (Đurek et al., 2018; Kane et al., 2017). Today, new media have become not just an attractive addition, but a full-fledged learning and teaching environment. The COVID-19 pandemic has accelerated the process of digitisation of education, revealing the strengths and weaknesses of school systems around the world. Thus, the presented research results become another micro-stage for building and redefining the processes connected to the pedagogy of media and information society.
